# Comparison of the ruminal and fecal microbiotas in beef calves supplemented or not with concentrate

**DOI:** 10.1371/journal.pone.0231533

**Published:** 2020-04-13

**Authors:** Jeferson M. Lourenco, Troy J. Kieran, Darren S. Seidel, Travis C. Glenn, Magali F. da Silveira, Todd R. Callaway, R. Lawton Stewart

**Affiliations:** 1 Department of Animal and Dairy Science, University of Georgia, Athens, GA, United States of America; 2 Department of Environmental Health Science, University of Georgia, Athens, GA, United States of America; 3 Universidade Tecnológica Federal do Paraná, Dois Vizinhos, PR, Brazil; Universidade Federal de Viçosa, BRAZIL

## Abstract

Most of the research efforts involving the bovine gastrointestinal microbiota have focused on cattle’s forestomach, particularly the rumen, so information concerning the bovine fecal microbiota is more scarce, especially in young beef cattle. The present study was performed to evaluate the ruminal and fecal microbiotas of beef calves as they reached the end of their nursing phase. A total of 18 Angus cow/calf pairs were selected and assigned to one of two treatment groups for the last 92 days of the calves’ nursing period, as follows: 1) calves were supplemented with concentrate in a creep feeding system; or 2) control group with no supplementation of calves. After 92 days, ruminal and fecal samples were individually obtained from calves in both groups, and their microbiotas were evaluated using 16S rRNA gene sequencing. Ruminal samples were predominated by *Prevotella* (18 to 23% of the total bacterial abundance), regardless if calves received supplementation or not; however, in the feces, *Prevotella* was only the seventh most abundant genus (0.6 to 2.1% of total bacterial abundance). Both the rumen (*P* = 0.01) and the feces (*P* = 0.05) of calves that received supplementation had greater abundance of *Firmicutes*. In addition, calves that were supplemented had lower abundance of *Fibrobacteres* (*P* = 0.03) in their rumens. Regardless if the calves were supplemented or not, Faith’s Phylogenetic Diversity index (*P* ≤ 0.007) and total concentration of short chain fatty acids (*P* < 0.001) were both greater in the rumen than in the feces of calves. In summary, the ruminal and fecal microbiotas of weanling beef calves were considerably distinct. Additionally, supplementation with creep feed caused some significant changes in the composition of the gastrointestinal microbiota of the calves, especially in the rumen, where supplementation caused an increase in *Firmicutes* and a decrease in abundance of *Fibrobacteres*.

## Introduction

The bovine gastrointestinal microbial population is composed of a consortium of microorganisms that exist in a symbiotic relationship with the host animal. Moreover, this gastrointestinal microbiota plays an important role in the host animal’s metabolism and homeostasis [1,2]. Next-generation DNA sequencing techniques have made determining nucleotide sequences increasingly affordable and has reached a point where personalized microbiology is a conceivable reality [3]. This massification of the technique is not different in the animal production industry, since the lower costs of DNA sequencing have encouraged their widespread use in the animal sciences.

In livestock, the gastrointestinal microbiota is associated with improved animal health, well-being, and growth performance [4,5,6]. Hence, generating microbial population data can inherently improve the understanding of host animal production dynamics. Moreover, a better understanding of this complex biological system can eventually allow livestock producers to improve animal health-related decisions, and increase performance in the long term while minimizing environmental impacts.

The microbiota living in the gut of cattle is very complex and is characterized by a high population density. This microbial population has been studied extensively in recent years [7,8,9,10]; however, the majority of the studies have focused on the microbiota that resides in the forestomach of cattle (rumen). As a consequence, information on the other segments of their gastrointestinal tract is more limited, particularly in beef calves in the nursery phase (from birth to 6–8 months of age). Therefore, this study aimed to assess both the ruminal and the fecal microbiotas of young beef calves raised under 2 distinct growing conditions–with or without concentrate supplementation via a creep feeding system–during the last third of the nursing phase.

## Materials and methods

This study was performed in strict accordance with the recommendations in the Guide for the Care and Use of Laboratory Animals of the National Institutes of Health. All procedures involving live animals were verified and approved by the University of Georgia’s Office of Animal Care and Use (Animal Use Protocol Number A2015 07–018-Y1-A0).

### Animals and management

The cattle herd used in this study was located at University of Georgia’s Eatonton Beef Research Unit, located in Eatonton, GA (33°24 N, 83°29 W). A group of 18 beef calves in their suckling phase (14 males and 4 females; body weight = 231 ± 20 kg; age = 155 ± 10 days-old), along with their respective dams, were assigned to one of two groups. The two groups were as uniform as possible by blocking for calf sex, body weight, and calf age. Calves in the two groups were treated for the last 92 days prior to weaning, as follows: 1) Group in which the calves were supplemented using a creep feeding system (i.e. system in which only the calves have access to the feed); or 2) Control group with no supplementation of the calves. The creep feed supplement was composed of corn, soybean meal, salt, molasses, and a vitamin-mineral premix (60.2, 25.0, 8.8, 1.1, and 4.9%, respectively). The group average daily intake of creep feed was 1.1 kg per calf. Calves had free access to the mineral mix that was offered to the cows, and their daily milk consumption was not assessed. The forage composition of the experimental paddocks was the same for both groups: The paddocks were composed of a combination of bermudagrass (*Cynodon dactylon*) and dallisgrass (*Paspalum dilatatum*). In order to access forage quality, two forage samples–one at the beginning and one at the end of the study–were obtained from each paddock. Growth performance of all calves was monitored by body weight on days 1, 53, and 92.

### Collection of ruminal and fecal samples

On day 92, 6 male calves were randomly selected from each treatment group and their ruminal contents were collected using esophageal tubing and vacuum. Approximately 20 mL of rumen fluid were obtained from each animal and were placed in sterile tubes. Fresh fecal samples were collected per rectum by digital palpation. Both the ruminal and fecal samples were flash frozen by immersion in liquid nitrogen immediately after collection and then stored at –20°C. Following sampling, all calves were weaned and moved to another part of the farm.

### Short chain fatty acid analyses

Ruminal and fecal samples were analyzed using the same protocol [11], the only difference being that the fecal samples had to be solubilized in water prior to being further processed (1 part of sample to 3 parts of water) [12]. Once prepared, samples were centrifuged at 10,000 x g for 10 minutes, and 1 mL of the resulting supernatant was pipetted into a new centrifuge tube, along with 0.2 mL of a metaphosphoric acid solution (25% wt/vol), and samples were frozen overnight. Samples were then thawed and centrifuged at 10,000 x g for 10 minutes. The supernatant was transferred into polypropylene tubes and mixed with ethyl acetate in a 2:1 ratio of ethyl acetate to supernatant. Tubes were vortexed for 15 seconds and allowed to settle for 5 minutes. Next, 0.5 mL of the top portion was transferred to screw-thread vials for analysis of short chain fatty acids (SCFA) in a Shimadzu GC-2010 Plus gas chromatograph (Shimadzu Corporation, Kyoto, Japan) equipped a flame ionization detector and a capillary column (Zebron ZB-FFAP; 30 m x 0.32 mm x 0.25 μm; Phenomenex Inc., Torrance, CA, USA). Sample injection volume was 1.0 μL, and helium was used as the carrier gas. Column temperature was initially set at 110°C and gradually increased to 200°C. Injector and detector temperatures were set at 250 and 350°C, respectively.

### DNA extraction and sequencing

Frozen ruminal and fecal samples were thawed, homogenized, and 0.5 mL of the ruminal, or 0.25 g of the fecal materials were processed according to the PowerSoil^®^ DNA Isolation Kit protocol (Version 11212013; MoBio Laboratories, Carlsbad, CA), which promotes bacterial lysis by a combination of mechanical and chemical methods. Purified DNA was eluted in 100 μL of molecular grade water and 30 μL of was transferred to a 96-well plate. Extracted DNA was amplified in replicates using two rounds of PCR, using the forward: S-D-Bact-0341-b-S-17 (5′-CCTACGGGNGGCWGCAG-3′); and reverse: S-D-Bact-0785-a-A-21 (5′-GACTACHVGGGTATCTAATCC-3′) 16S rRNA primer pair [13]. These primers were modified with the addition of Illumina TruSeq sequences and unique 8 + 12, forward and reverse, variable length barcodes [14,15]. Positive amplification was verified on a 1.5% agarose gel and equal amounts of each sample were pooled and cleaned with SPRI-beads (Thermo Fisher Scientific, Waltham, MA) with a 0.92:1 ratio. A second PCR amplification was performed on the pool in triplicate using iTru primers with 8 bp barcode indexes [16]. The complete Illumina library was purified with SPRI-beads (1:1 ratio) and sequenced on an Illumina MiSeq using a v3 600 cycle kit (Illumina, San Diego, CA) at the Georgia Genomics and Bioinformatics Core. Negative controls were used throughout extraction and PCR procedures, and used KAPA reagents (KAPA Biosystems, Wilmington, MA) as described in detail in a previous study [17].

### DNA data sequencing analysis

Sequencing data were demultiplexed by outer iTru indexes using bcl2fastq (Illumina, v1.8.4) and then by internal sample barcodes using Mr. Demuxy v1.2.0. Sequences were imported into Geneious v10.0.9 (Biomatters Limited, NJ), where we set paired-reads trimmed primers and low quality bases using default settings and a quality score of 0.001, and merged reads using the FLASH v1.2.9 plugin [18].

Data were exported from Geneious as FASTA files and further analyzed using the QIIME pipeline v1.9.1 [19]. Operational taxonomic units (OTUs) were clustered at 97% similarity, and cluster sequences were aligned to the Greengenes database (gg_13_8_otus; which is QIIME’s default database). Singleton OTUs, and OTUs whose representative sequences could not be aligned were excluded from the analysis. In addition, in order to eliminate sampling depth heterogeneity, alpha and beta-diversities were computed after sample sizes were standardized to 5,890 sequences. The computed alpha-diversity indexes were: Faith’s Phylogenetic Diversity, Shannon index, and the number of observed OTUs. Beta-diversity was computed using the weighted UniFrac distance matrix and plotted in a 3-dimension plot. Predictions of the functional composition of the ruminal and fecal metagenomes were also performed using the 16S rRNA gene as a marker and the PICRUSt computational approach [20]. Predicted metagenome functions were carried out using third-level KEGG pathways.

### Statistical analysis

Statistical analyses were performed using the software R v3.3.3 (R Foundation for Statistical Computing, Vienna, Austria), and Minitab^®^ v18.1. The software R was used for analysis of beta-diversity (packages ape and vegan), and Minitab^®^ for the other analyses. Comparisons between ruminal and fecal samples within the same group (i.e. supplemented or non-supplemented calves) were performed using paired t-tests. Comparisons between supplemented and non-supplemented calves within the same environment (rumen or feces) were carried out by Student’s t-test. Analysis of SCFA concentrations were carried out by ANOVA using the supplementation status and the type of sample analyzed as factors, along with their interactions. The differences in beta-diversity were accessed using two-sample t-tests, and the resulting *P*-values were corrected by Bonferroni’s method for multiple comparisons. For all the statistical tests used, results were considered significant at *P* ≤ 0.05, and were treated as trends when 0.05 < *P* < 0.10.

## Results and discussion

### Information on the microbiota of beef calves

Only a limited number of studies have performed direct comparisons between the microbiotas of the rumen and the feces in the same animals [21,22,23,24,25]. Additionally, this kind of comparison in beef calves is particularly scarce. Another limitation found in some of those studies is the sample size. For instance, the studies performed by de Oliveira et al. [22] and Durso et al. [24] analyzed the microbiota of only one adult animal. Consequently, although these two studies analyzed a wide variety of samples which comprised the entire bovine gastrointestinal tract (i.e. forestomach, small intestine, large intestine, and feces), the reliability of their results is compromised by the size of their samples. Furthermore, the two animals used in those two studies were adults, whereas the ones used in the present study were still in their nursing stage and presumably still transitioning to being fully functional ruminants. Despite the aforementioned limitations and differences with our study, both de Oliveira et al. [22] and Durso et al. [24] found differences in microbial diversity when a direct comparison between the rumen and feces of steers was performed. More specifically, those authors observed greater diversity in the rumen compared to the fecal material, so their findings are in line with the ones reported in the present study.

Another study that evaluated both the fecal and ruminal environments was carried out by Callaway and collaborators [21]; however, in that study, authors sought to determine the effects of feeding increasing levels (up to 40% of the daily intake) of dried distillers grains into cattle diets, and to determine if inclusion of this feedstuff affected foodborne pathogen populations indirectly by altering the microbiota. Because of those very specific goals, no indices of microbial diversity were presented in their research, nor were any direct contrasts between the fecal and ruminal microbiotas. Additionally, again, cattle used in their research were all adults (>14 months of age). Conversely, one study that did utilize young calves was carried out by Meale et al. [23]; however, this study used Holstein dairy calves rather than beef calves, and dairy calves are typically reared under very different conditions compared to beef calves. For instance, dairy calves are normally weaned at earlier ages (< 3 months) compared to beef calves (7–9 months of age). Thus, another difference between our study and the one performed by Meale et al. [23] is the age of the calves. Those researchers weaned their Holstein calves on day 49 of age, and collected the last sample for their study when calves were 54 days-old. In contrast, the Angus beef calves used in our study were weaned at day 247 of age, which was the same day our samples were taken. Therefore, due to different reasons, studies performing specific comparisons between the ruminal and fecal microbiotas in weanling beef calves are scarce.

### Microbial diversity and production of short chain fatty acids

**Fig 1:**
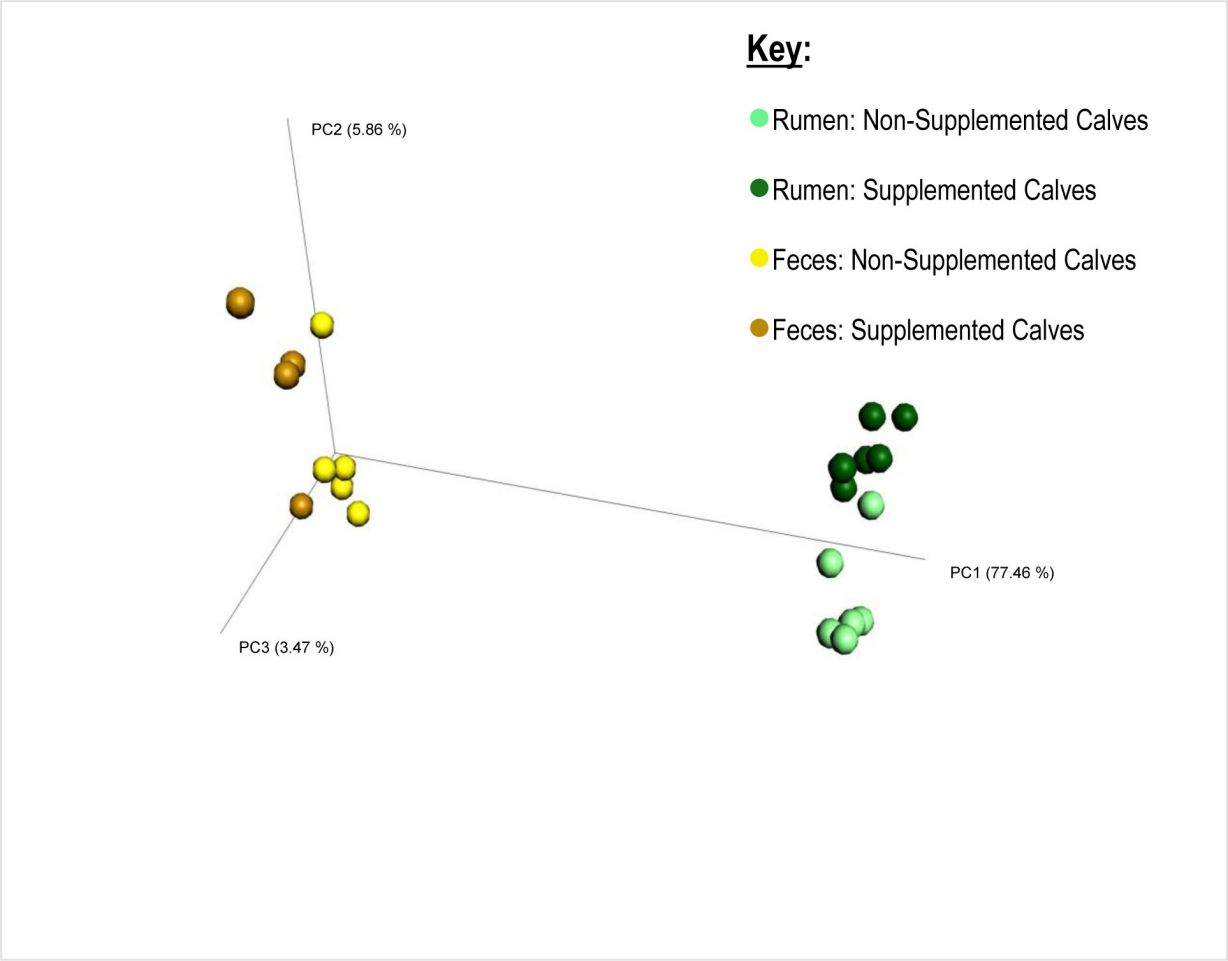
Beta-diversity (based on weighted UniFrac distance matrix) of the ruminal and fecal environments from supplemented and non-supplemented beef calves. (Bonferroni-corrected *P*-value for differences between groups was < 0.00001; for fecal samples of supplemented versus non-supplemented calves was 0.99; and for ruminal samples of supplemented versus non-supplemented calves was 0.001).

**Fig 2:**
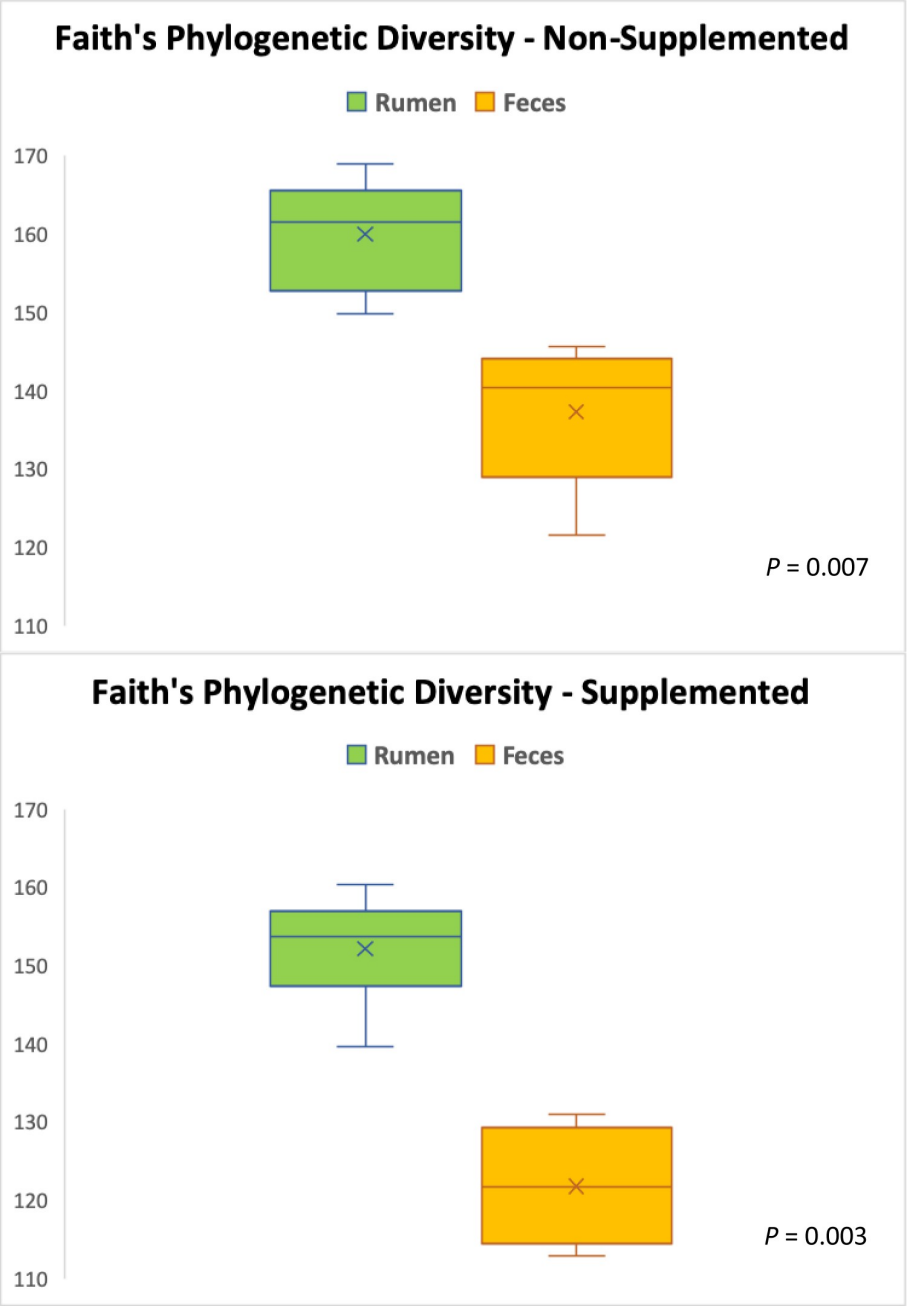
Comparison of Faith’s Phylogenetic Diversity index between the rumen and fecal microbiotas in supplemented and non-supplemented beef calves. (Diversity was greater in the rumen than it was in the feces: *P* = 0.007 and 0.003 for non-supplemented and supplemented calves, respectively).

**Fig 3:**
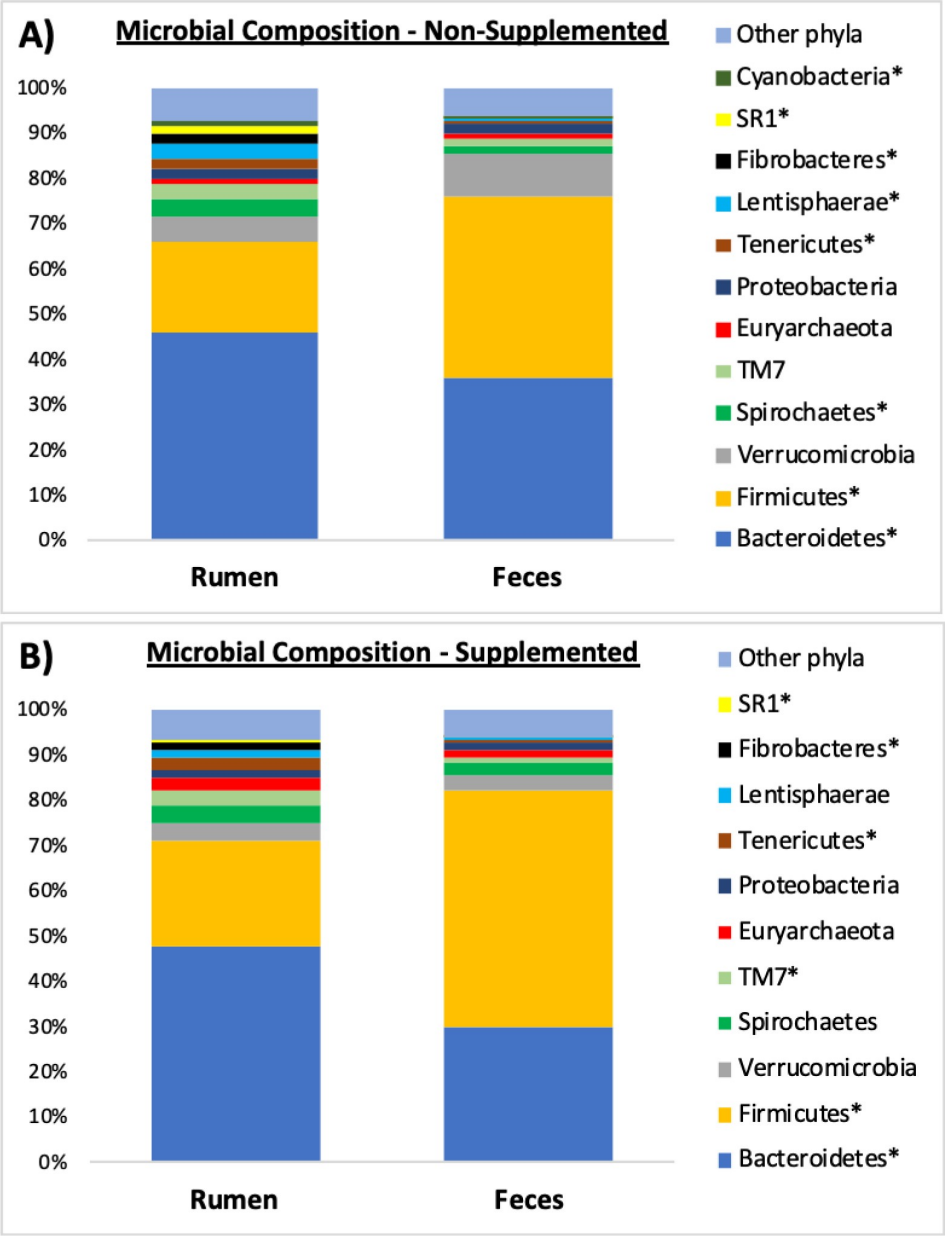
Comparison of the microbial composition (phylum level) found in the rumen and feces of non-supplemented (A); and supplemented beef calves (B). Only phyla with relative abundances ≥ 1% in at least one sample type are shown. * Indicates a *P*-value ≤ 0.05 for the contrast: rumen versus feces.

After all the quality control steps, the resulting samples yielded a total of 239,601 sequences, with an average of 11,410 sequences per sample. The number of sequences in the used samples ranged from 5,892 to 13,761, and they were rarefied to a sequencing depth of 5,890 sequences per sample. Regardless if the calves received supplementation or not, their fecal and ruminal microbiotas were very different (Figs 1–3). Beta-diversity information (Fig 1) allows a clear visualization of this: while all the fecal samples (both from supplemented and non-supplemented calves) tended to cluster together, the ruminal samples were significantly (*P* < 0.0001) separated from them, and gathered on the other end of the spectrum, regardless of the supplementation status. Faith’s Phylogenetic Diversity index (Fig 2) was similar to the beta-diversity findings and further indicated that the ruminal and fecal microbiotas were significantly distinct. Moreover, this index revealed that microbial diversity was greater (*P* ≤ 0.007) in the ruminal microbiota compared with the fecal microbiota of calves, regardless if they were supplemented or not. A greater microbial diversity in the rumen compared to the feces makes ecological sense, given the greater availability of nutrients that exists in the rumen compared to the large intestine, since most of the nutrients in feedstuffs are broken down and absorbed before reaching the large intestine [26].

There were no interactions (*P* ≥ 0.43) between the inclusion of supplement in calves’ diet and the type of sample (rumen or feces) on SCFA (Table 1); however, a trend (*P* = 0.10) was observed for supplemented calves to have more SCFA both in their ruminal and fecal contents, compared to non-supplemented animals. Furthermore, overall, the greatest contrasts concerning concentration of SCFA were observed due to the distinction between the types of sample analyzed (feces or rumen contents). Except for isobutyrate and valerate, all of the other SCFA (and total SCFA concentration) were higher (*P* ≤ 0.01) in the ruminal environment, compared to the fecal environment.

**Table 1:** Effect of supplementation on production of short chain fatty acids (SCFA) in 2 sample types (rumen or feces) obtained from beef calves.

	Non-Supplemented		Supplemented		*P*-value^1^		
SCFA (m*M*)	Rumen	Feces	Rumen	Feces	Supplement	Sample Type	Interaction
Acetate	63.17	25.11	67.59	33.47	0.13	**< 0.001**	0.63
Propionate	10.51	4.04	11.66	5.69	**0.05**	**< 0.001**	0.71
Butyrate	6.29	0.77	7.90	1.64	**0.04**	**< 0.001**	0.51
Isobutyrate	0.66	0.60	0.60	0.52	0.24	0.21	0.85
Valerate	0.45	0.32	0.53	0.52	0.13	0.44	0.53
Isovalerate	0.77	0.41	0.73	0.32	0.34	**< 0.001**	0.73
Caproate	0.30	0.08	0.40	0.00	0.96	**0.01**	0.43
Total SCFA	82.16	31.35	89.41	42.16	0.10	**< 0.001**	0.74

^1^ *P*-values calculated for the contrast between the presence of supplement in the diet, the type of sample (rumen or feces), and their interaction.

Total production of SCFA is in agreement with our calculated microbial diversity indexes, given that a greater concentration (*P* < 0.001) of total SCFA was found in the rumen compared to the feces, whether calves were supplemented or not. In the gastrointestinal tract, SCFA are the result of microbial fermentation of organic matter, which is a process that constantly occur in the rumen and large intestine [27,28]. Even though this fermentation occurs at both of those locations, the greater SCFA concentrations observed in the rumen when compared to the feces further indicates that greater microbial activity took place in the calves’ forestomach. In fact, for all of the individual SCFA quantified in the present study, the concentrations were numerically greater in the rumen than in the feces of the calves, and this remained true for both groups of calves–supplemented and non-supplemented.

When comparing the concentration of each SCFA individually, it can be noticed that supplementation of the calves did not have a significant impact on the majority of them, except for propionate (*P* = 0.05) and butyrate (*P* = 0.04), which were both found in greater concentrations in the gastrointestinal tract of calves that were supplemented. Propionate is an important glucose precursor in ruminants [29], and greater concentrations of this SCFA have been found to be positively correlated with body weight gain in cattle [30]. In addition, propionate has stimulatory effects on the development of the ruminal mucosa in calves [31]. Likewise, it has been established that butyrate stimulates the development of the rumen epithelium like no other SCFA [32,33]. Therefore, although we did not assess the development of the ruminal epithelial tissue in this study, the greater quantities of propionate and butyrate found in the gastrointestinal tract of supplemented calves likely contributed to a better development of the ruminal epithelial tissue in this group of calves, compared to the control group.

### Composition of microbiotas at the phylum level

**Fig 4:**
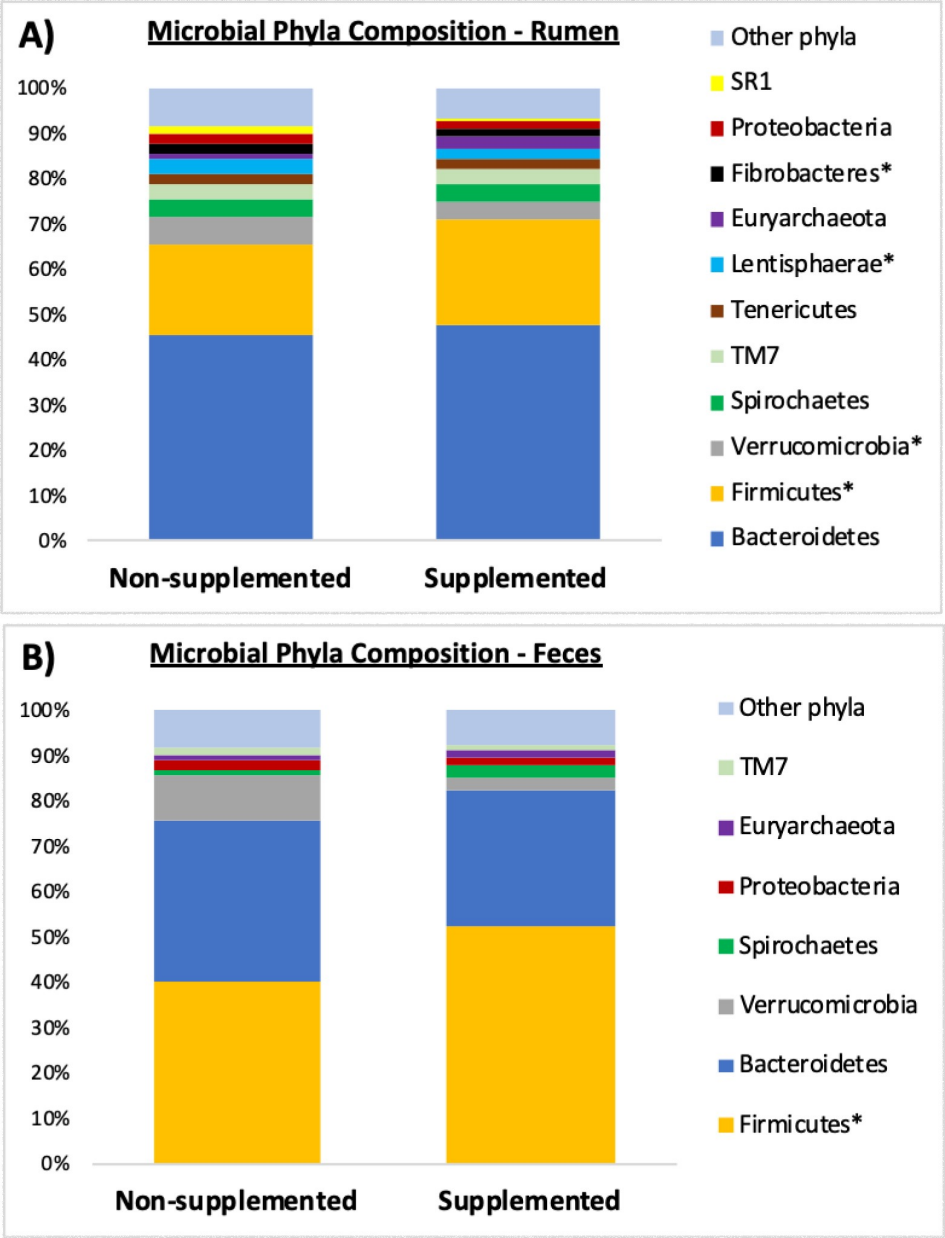
Microbial phyla composition found in the rumen (A) and feces (B) of non-supplemented and supplemented beef calves. Only phyla with relative abundances ≥ 1% in at least one sample type are shown. * Indicates a *P*-value ≤ 0.05 for the contrast: non-supplemented versus supplemented.

Microbial composition at the phylum level for the rumen and feces of the calves are shown in Figs 3 and 4. Of the 12 main phyla comprising the microbiota of non-supplemented calves (Fig 3A), 8 had significantly different (*P* ≤ 0.05) abundances between the rumen and the feces: *Cyanobacteria*, SR1, *Fibrobacteres*, *Lentisphaerae*, *Tenericutes*, *Spirochaetes*, *Firmicutes*, and *Bacteroidetes*. Of the 11 main phyla detected in the microbiota of supplemented calves (Fig 3B), 6 were found at different (*P* ≤ 0.05) abundances when comparing the ruminal and fecal environments of supplemented calves, namely: SR1, *Fibrobacteres*, *Tenericutes*, TM7, *Firmicutes*, and *Bacteroidetes*. Comparison of phyla abundance between supplemented and non-supplemented calves within the rumen is shown in Fig 4A, and within the feces in Fig 4B. In the rumen, *Fibrobacteres*, *Lentisphaerae* and *Verrucomicrobia* were detected at lower abundances (*P* ≤ 0.03) in calves that were supplemented, whereas *Firmicutes* were found at greater abundance (*P* = 0.01) in creep fed calves. When comparing just the fecal samples, there was a trend (*P* = 0.06) for abundance of *Verrucomicrobia* to be lower in the group of calves that were supplemented, and a significant increase (*P* = 0.05) in the abundance of *Firmicutes* in that group.

When analyzing just the ruminal contents, de Oliveira et al. [22] found that the phyla *Bacteroidetes* was the most prevalent followed by *Firmicutes*. Conversely, in the feces, those authors found that *Firmicutes* composed a greater percentage of the population, followed by *Bacteroidetes*. The present study found similar results concerning *Firmicutes* and *Bacteroidetes*. Similar to the adult animal described by de Oliveira et al. [22], feces from the young calves in our study were dominated by *Firmicutes* (53% and 40% for supplemented and control calves, respectively), followed by *Bacteroidetes* (30% and 36%). Concerning the ruminal samples from our calves, *Bacteroidetes* were the most abundant phylum (47% and 46%), followed by *Firmicutes* (24% and 20% for supplemented and control calves, respectively). Thus, these two bacterial phyla were the most abundant both in the feces and in the rumen of our calves, regardless of supplementation. In recently-weaned Holstein dairy calves, Meale et al. [23] also detected a preeminence of *Bacteroidetes* and *Firmicutes*; however, in their study, the abundance of *Bacteroidetes* was greatest in both the fecal and ruminal environments. Such contrasts between our study and [23] are likely due to a combination of different factors such as feeding management, diets, and age of the animals. While the dairy calves in [23] were fed milk-replacer twice a day, our beef calves had free access to their mother’s milk throughout the day. Moreover, in our study, calves grazed fresh forages (bermudagrass and dallisgrass), whereas in their study calves were offered chopped straw as their roughage source. Lastly, the age of the calves was also substantially different: while the last sample used in their study was collected when calves were 54 days-old, our samples were collected when calves were 247 days-old.

Beyond the two main phyla discussed above, other significant contrasts were detected in the rumen (but not in the feces) between calves that were supplemented or not. Noteworthy, abundance of *Fibrobacteres* in the rumen dropped from 2.3% in the control group to 1.5% in the group that was supplemented. *Fibrobacteres* are recognized as major bacterial degraders of cellulose [34], thus, a decrease in the abundance of this phylum in the rumen of calves that received an extra load of readily-degradable carbohydrates (e.g. starch) in the form of supplement seems reasonable when compared to the control calves. Differently from the creep fed group, calves in the control group only had access to grass as their source of solid feed. Given that grasses have a greater fiber content relative to concentrates, a higher presence of fibrolytic bacteria such as *Fibrobacteres* in the rumen of calves from the control group seems to be logical.

### Composition of microbiotas at the genus level

The lowest taxonomic level at which the samples in this study were classified was the genus level, and the 10 most abundant bacterial genera detected in the rumen and feces of the calves are presented in Tables 2 and 3, respectively. In the ruminal environment, abundance of *Prevotella* and *Fibrobacter* significantly varied between supplemented and non-supplemented animals. More specifically, supplemented calves had greater (*P* = 0.01) abundance of *Prevotella*, and lower (*P* = 0.03) abundance of *Fibrobacter* compared to non-supplemented calves. In the fecal samples, of the top 10 bacterial genera identified, *Prevotella* and *Lactobacillus* were significantly altered due to the inclusion of supplement in the diet of calves: while abundance of *Prevotella* was lower (*P* < 0.01) in the feces of creep fed calves, abundance of *Lactobacillus* was greater (*P* = 0.01) in this group of animals.

**Table 2:** Top 10 bacterial genera identified in the rumen of beef calves that were supplemented or not in a creep feeding system.

	Average Relative Abundance (%)			
Bacterial Genera	Overall Abundance	Non-Supplemented Calves	Supplemented Calves	*P*-value^1^
*Prevotella*	20.75	18.28	23.22	**0.01**
*Treponema*	3.76	3.72	3.79	0.90
CF231	2.60	2.64	2.57	0.87
RFN20	2.12	2.24	2.00	0.57
*Fibrobacter*	1.92	2.30	1.53	**0.03**
*Methanobrevibacter*	1.72	0.81	2.62	0.06
*Anaeroplasma*	1.27	1.34	1.19	0.52
*Pseudobutyrivibrio*	1.23	1.09	1.36	0.17
YRC22	1.17	1.18	1.16	0.87
*Ruminococcus*	1.12	0.93	1.31	0.18

^1^ *P*-value for the contrast between supplemented and non-supplemented calves.

**Table 3:** Top 10 bacterial genera identified in the feces of beef calves that were supplemented or not in a creep feeding system.

	Average Relative Abundance (%)			
Bacterial Genera	Overall Abundance	Non-Supplemented Calves	Supplemented Calves	*P*-value^1^
5-7N15	7.07	8.39	5.74	0.19
*Akkermansia*	5.74	8.88	2.61	0.10
*Dorea*	3.99	1.97	6.00	0.26
*Ruminococcus*	3.00	0.37	5.63	0.21
*Treponema*	2.04	1.28	2.80	0.25
CF231	1.94	1.21	2.68	0.09
*Prevotella*	1.35	2.11	0.59	**< 0.01**
*Lactobacillus*	1.13	0.55	1.71	**0.01**
*Methanobrevibacter*	0.97	0.91	1.04	0.71
*Oscillospira*	0.91	1.04	0.78	0.25

^1^ *P*-value for the contrast between supplemented and non-supplemented calves.

As stated before, *Prevotella* was the most abundant genus detected in the ruminal samples, and its average abundance was greater in the creep fed calves. Previous research from our group [17] also observed that the presence of *Prevotella* was increased in the rumen of creep fed calves. That study also identified a positive correlation between abundance of *Prevotella* and calf average daily gains. The genus *Prevotella* contains many metabolically active species, which play a wide variety of biological roles. For instance, *Prevotella ruminicola* has been regarded as a widely adapted species that can utilize starch and a range of other soluble sugars, and in some instances it can break down complex polysaccharides like hemicellulose and pectin [35]. However, although *Prevotella* have this capability of breaking down different carbohydrates, one of their most significant roles is in the protein and peptide breakdown [35,36]. In fact, a comparison of 14 different species of ruminal bacteria revealed that *Prevotella ruminicola* was the most important single species in peptide breakdown in the rumen [36]. Thus being, the greater abundance of *Prevotella* in the ruminal samples of our creep fed calves was likely a response to the greater quantity of readily available carbohydrates and protein in the rumen of the supplemented calves.

### Calf body weight gain and microbiota predicted functional profile

**Fig 5:**
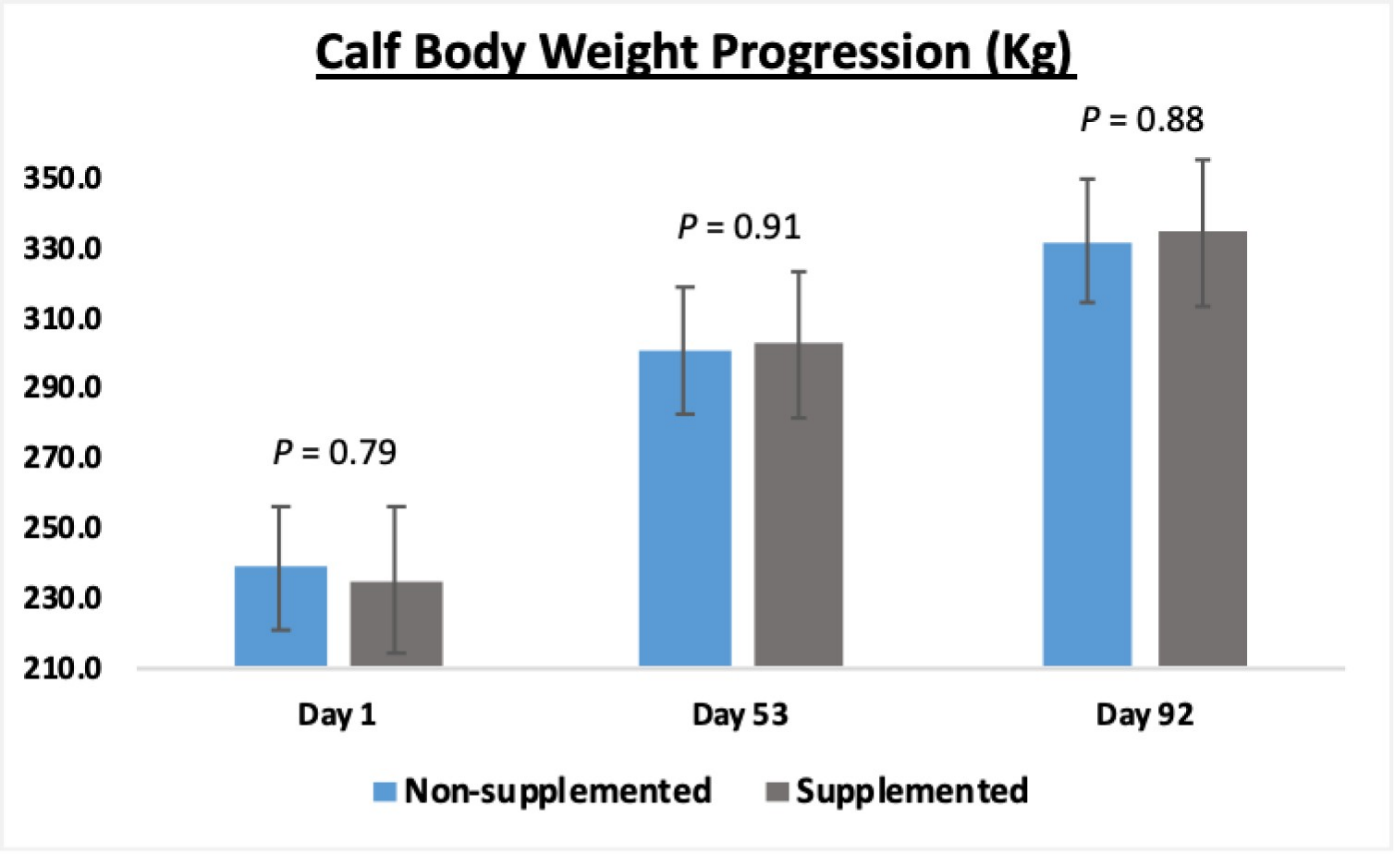
Calf body weight progression during the 92-day trial in which 5-month-old beef calves were supplemented or not in a creep feed system. No significant differences in their body weights due to supplementation were detected at the beginning, middle, and end of the trial (*P* ≥ 0.79).

Supplementing the beef calves using creep feed during the last 92 days prior to their weaning did not increase (*P* ≥ 0.79) their body weights, nor their rates of weight gain (Fig 5). In a year with abundant high-quality pastures, such growth improvements were simply numerical, and the final body weights observed for non-supplemented and supplemented calves were 331.7 and 334.4 kg, respectively. This lack of difference was likely due to the quality of the forage on which the cow/calf pairs were housed. Not only did the high-quality grass provide good forage for the calves, but it also supported optimum milk production for the cows, which in turn resulted in high calf body weight gain in both groups, regardless of supplementation. This is evidenced by the fact that non-supplemented calves gained a total of 93.4 kg during the 92-day trial, while supplemented calves gained 99.6 kg, totalizing a difference of only 6.2 kg in body weight gain. Thus, similar to the results reported by Aguiar et al. [37] and Reis et al. [38], the creep feed supplementation utilized in this study added little value to calf performance.

**Fig 6:**
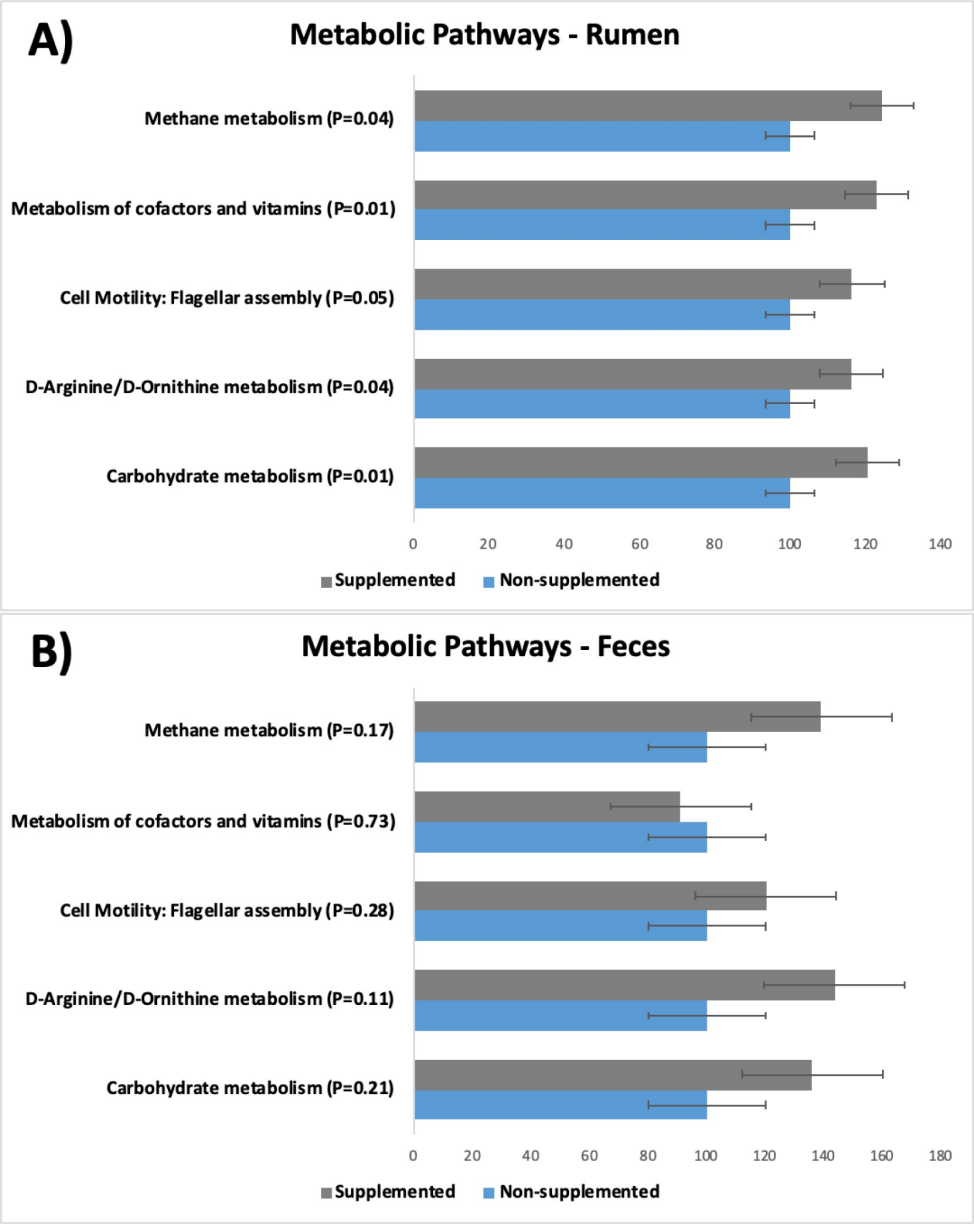
Predicted metabolic pathways (third-level KEGG pathways) computed for calves that were supplemented or not. Values are presented as percentage of expression relative to the non-supplemented (control) group for: A) Ruminal samples; and B) Fecal samples.

Predictive metagenomics identified five KEGG pathways which had different expressions (*P* ≤ 0.05) in the rumen of supplemented calves in comparison to the ones that were not supplemented (Fig 6). However, the same pathways were not differently expressed (*P* ≥ 0.11) in the two groups of calves when analyzing the fecal samples. The lack of differences in the feces was partially due to the greater variation observed in those samples, compared to the ones obtained from the rumen.

Although concentrate supplementation caused no differences in body weight gain in this study, some differences in the calves’ microbiotas were observed, and therefore, differences in the bacterial activity and consequently different pathway expressions were anticipated. Even though the pathways shown in the present study were obtained using computational biology [20] and were not measured directly, the results make biological sense. For instance, a greater expression of a metabolic pathway associated with cofactors and vitamins in the rumen of calves that were creep fed is not unexpected, since it has been established for many decades that synthesis of several vitamins (especially vitamin B complex) occur in the rumen [39,40]. Furthermore, Schwab et al. [41] found that decreasing the level of forage in diet of cows increased synthesis of pyridoxine, folic acid, and vitamin B_12_.

Greater expression of pathways related with D-arginine and D-ornithine metabolism were also observed in the rumen of supplemented calves. D-ornithine has been shown to be a constituent of bacterial cell wall of gram positive bacilli [42]. D-arginine may be part of specific bacterial secretions that allow some bacteria to thrive in polymicrobial communities, since these secretions are toxic to other microorganisms [43]. So greater metabolism of D-amino acids is compatible with the greater microbial activity that likely took place in the rumen of creep fed calves, as the amount of total SCFA indicated. Lastly, greater expression of pathways involved in the metabolism of carbohydrates and flagellar assembly further corroborate with the perception of greater microbial activity in the rumen of supplemented calves, since carbohydrates are the main component of the supplement, and more flagellar assembly have to occur in an environment with more bacteria, given that many species move using flagella [44].

## Conclusions

Collectively, our results demonstrated that the ruminal and fecal microbiotas of beef calves were very different, regardless if the calves were supplemented or not during the nursing phase. Alpha and beta-diversities, the individual microbial compositions (at both the phylum and genus levels), and the quantified microbial metabolites (SCFA) agreed that the fecal and ruminal environments were dissimilar, despite the fact that some bacterial groups were present in both of those environments. Furthermore, despite the lack of response on calf growth, supplementing the calves for 92 days significantly increased the ruminal abundance of *Prevotella* and decreased the ruminal population of *Fibrobacter*. In the fecal environment however, supplementation of calves decreased abundance of *Prevotella* and increased population of *Lactobacillus*. Lastly, it was observed that supplementing the calves with creep feed significantly altered the expression of some microbial metabolic pathways in the rumen, but not in the feces, suggesting that supplementation had an impact primarily in their ruminal microbiotas. Further research is needed to understand all the effects of supplementing calves during the nursing phase on their gastrointestinal microbiotas, and the subsequent impacts on later growth, production efficiency, and carcass quality of the calves as they reach maturity.

## Supporting information

S1 Table Average chemical composition of the forage and mineral supplement offered to the cow-calf pairs during the study (dry matter basis). (DOCX)

S1 Fig Number of OTUs found in the rumen and feces of the supplemented and non-supplemented calves. (DOCX)

S2 Fig Shannon Diversity Index for the ruminal and fecal environments in supplemented and non-supplemented calves. (DOCX)
