# Supplementary material for: Comparison of the ruminal and fecal microbiotas in beef calves supplemented or not with concentrate
Source: PLoS One. 2020 Apr 13;15(4):e0231533. doi: 10.1371/journal.pone.0231533 (PMC7153887; doi:10.1371/journal.pone.0231533)
Supplement: S1 Table — (DOCX) [file pone.0231533.s001.docx]

| **S1 Table.** Average chemical composition of the forage and mineral supplement offered to the cow-calf pairs during the study (dry matter basis)^1^. | | |
| --- | --- | --- |
|  |  |  |
| **Item** | **Feedstuff** | |
|  | **Forage** | **Mineral Supplement** |
| Crude protein (%) | 11.60 | - |
| Neutral detergent fiber (%) | 66.96 | - |
| Acid detergent fiber (%) | 38.70 | - |
| Ether extract (%) | 2.19 | - |
| Non-fiber carbohydrates (%) | 10.00 | - |
| Net energy for maintenance (Mcal/kg) | 1.21 | - |
| Net energy for gain (Mcal/kg) | 0.64 | - |
| Calcium (%) | 0.43 | 13.20 |
| Phosphorus (%) | 0.33 | 6.10 |
| Magnesium (%) | 0.16 | 2.60 |
| Sodium (%) | 0.02 | 7.80 |
| ^1^ Cumberland Valley Analytical Services (Hagerstown, MD). | | |
