# Supplementary material for: Comparison of the ruminal and fecal microbiotas in beef calves supplemented or not with concentrate
Source: PLoS One. 2020 Apr 13;15(4):e0231533. doi: 10.1371/journal.pone.0231533 (PMC7153887; doi:10.1371/journal.pone.0231533)
Supplement: S1 Fig — (DOCX) [file pone.0231533.s002.docx]

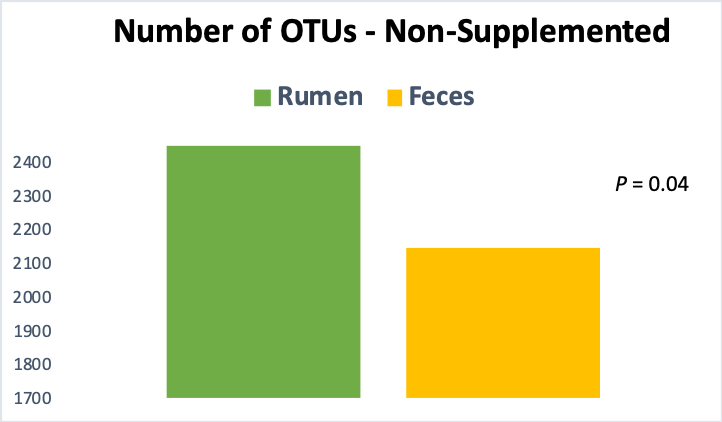

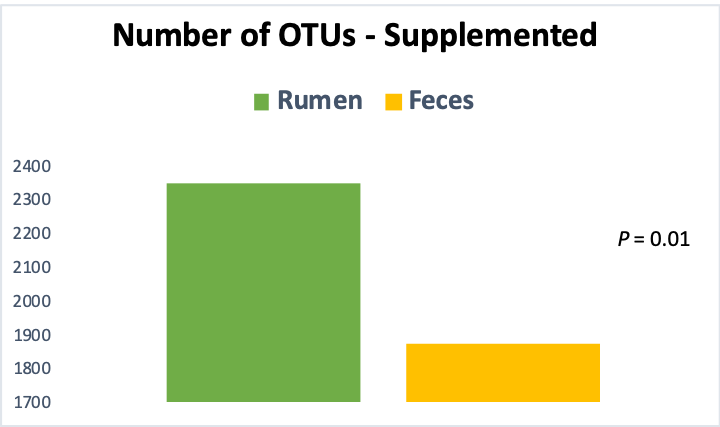


**S1 Fig. Number of OTUs found in the rumen and feces of the supplemented and non-supplemented calves.**
