# Supplementary material for: Comparison of the ruminal and fecal microbiotas in beef calves supplemented or not with concentrate
Source: PLoS One. 2020 Apr 13;15(4):e0231533. doi: 10.1371/journal.pone.0231533 (PMC7153887; doi:10.1371/journal.pone.0231533)
Supplement: S2 Fig — (DOCX) [file pone.0231533.s003.docx]

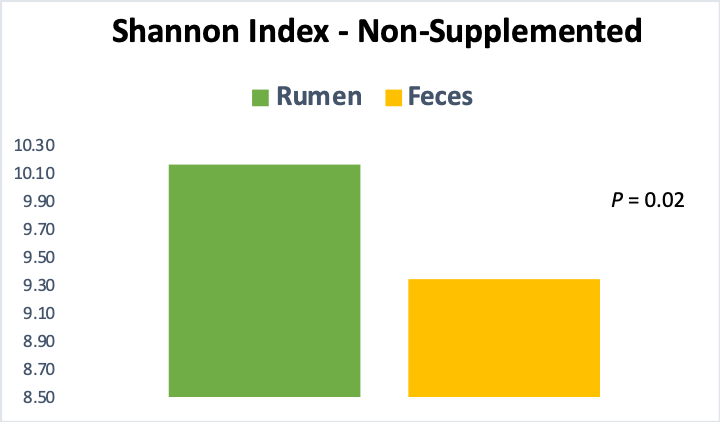

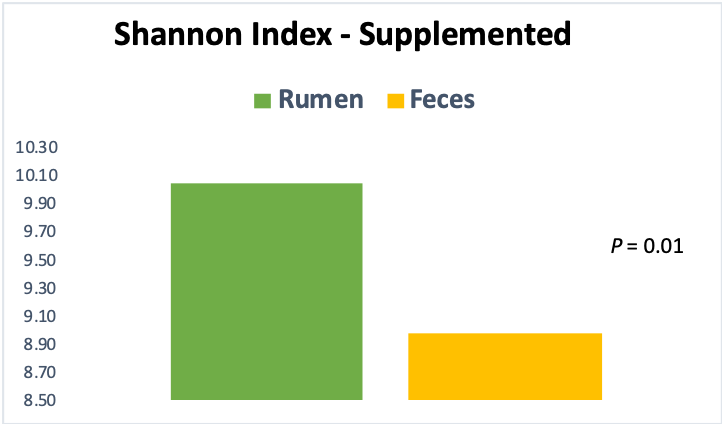


**S2 Fig. Shannon Diversity Index for the ruminal and fecal environments in supplemented and non-supplemented calves.**
